# A Brief Review of Chelators for Radiolabeling Oligomers

**DOI:** 10.3390/ma3053204

**Published:** 2010-05-14

**Authors:** Yuxia Liu, Guozheng Liu, Donald J. Hnatowich

**Affiliations:** Division of Nuclear Medicine, Department of Radiology, University of Massachusetts Medical School, Worcester, MA 01655, USA; E-Mails: yuxia.liu@umassmed.edu (Y.L.); donald.hnatowich@umassmed.edu (D.J.H.)

**Keywords:** oligomers, DNA analog, chelator, radionuclide

## Abstract

The chemical modification of oligomers such as DNA, PNA, MORF, LNA to attach radionuclides for nuclear imaging and radiotherapy applications has become a field rich in innovation as older methods are improved and new methods are introduced. This review intends to provide a brief overview of several chelators currently in use for the labeling of oligomers with metallic radionuclides such as ^99m^Tc, ^111^In and ^188^Re. While DNA and its analogs have been radiolabeled with important radionuclides of nonmetals such as ^32^P, ^35^S, ^14^C, ^18^F and ^125^I, the labeling methods for these isotopes involve covalent chemistry that is quite distinct from the coordinate-covalent chelation chemistry described herein. In this review, we provide a summary of the several chelators that have been covalently conjugated to oligomers for the purpose of radiolabeling with metallic radionuclides by chelation and including details on the conjugation, the choice of radionuclides and labeling methods.

## 1. Introduction

Advances in nuclear imaging require adequate methods for labeling biologicals such as DNA and its analogs (referred to herein as oligomers) with a variety of radionuclides, those that emit gamma rays for noninvasive imaging and those emit betas, alphas, Auger electrons, *etc.* for radiotherapy. Compared to other imaging modalities such as MRI, CT, ultrasound and optical, nuclear imaging offers extremely high sensitivity capable of detecting contrast agents at pM concentrations or lower [1]. Compared to external beam or brachytherapy, radiation therapy with internally administered radionuclides can offer superb dose distribution [2]. Oligomers are unique in their property of hybridization to their complement and this property has been exploited in the development of novel radiopharmaceuticals for nuclear imaging and radiotherapy by pretargeting, by antisense localization and by aptamer-mediated approaches [3,4,5]. While the use of unlabeled oligomers in medicine has been extensively reported over the past several decades, much less is presently in the literature regarding methods of labeling these oligomers with radionuclides, especially metallic radionuclides. Radionuclides such as ^14^C, ^11^C, ^14^N, ^35^S, ^3^H and ^32^P have been used to label biologicals including oligomers, often by isotope substitution (e.g., ^11^C for ^12^C, ^3^H for ^1^H). While labeling by isotope substitution essentially guarantees that the properties of the biological will not have been altered, the labeling process is usually nontrivial. With few exceptions, biologicals cannot be radiolabeled with metallic radionuclides in this manner but require the preliminary covalent attachment of a chelator, a chemical structure capable of binding a metal in a claw-like fashion with two or more bonds. Since chelators are usually large molecules, in contrast to nonmetals, labeling with metals is much more likely to alter biological properties. However, an important advantage of labeling by chelation is that the labeling itself can be extremely simple to the point where kit formulations are possible. This contribution is intended to provide a brief description of several chelators that have been successfully used to radiolabeled oligomers. The coverage is not intended to be comprehensive. Rather we hope merely to raise awareness of what has been done in the recent past and of some of the problems that have been successfully addressed.

## 2. Oligomers

**Figure 1:**
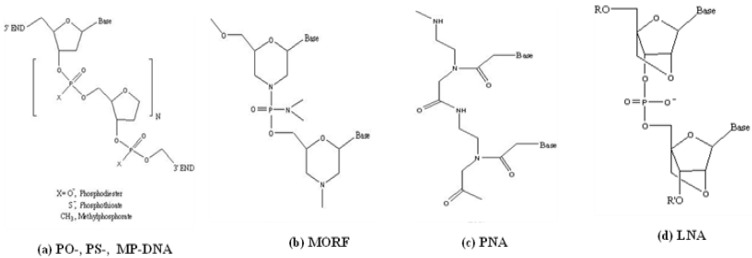
Chemical structures of DNAs and several important analogs. **(a)** phosphodiester (PO), phosphorothioate (PS) and methylphosphonate (MP) DNAs; **(b)** phosphorodiamidate morpholino (MORF); **(c)** peptide nucleic acids (PNA); **(d)** locked nucleic acids (LNA).

The term oligomers as used herein refers to oligonucleotides such as DNAs and RNAs and includes analogs that are not polynucleotides (such as PNAs, MORFs and LNAs). To overcome the instability to nucleases of native DNA and RNAs with phosphodiester (PO) backbones, several families of synthetic analogs have become commercially available, including phosphorothioate (PS) DNAs [6], phosphorodiamidate morpholino oligomers (MORF) [7], peptide nucleic acids (PNA) [8] and locked nucleic acid (LNA) [9,10] as shown in Figure 1. The modified backbones in each of these synthetic DNA analogs have stabilized the oligomers against nuclease hydrolysis while still permitting similar, in some cases increased, affinities for their complement.

## 3. Metallic Radionuclides

Theoretically, almost any radionuclide can be chemically attached to oligomers, but the choice will depend upon the application. Although justification of a particular choice is usually not now included in published reports, implicit are those factors extensively discussed decades ago [11,12,13], including (1) decay type, (2) physical half life, (3) availability and (4) ease of labeling. For example: radionuclides decaying by short-range high LET emissions such as betas, alphas and Auger electrons are highly cytotoxic and used to label biologicals designed for radiotherapeutic purposes, while diagnostic imaging agents require radionuclides that are relatively low in cytotoxicity but decay with imaginable emissions, either gammas for planar and Single Photon Computerized Emission Tomography (SPECT) imaging or annihilation photons for Positron Emission Tomography (PET). In general, radionuclides with short physical half lives are preferred for imaging to minimize the radiation exposure to subjects. However, too short a half life may not provide sufficient time for radiolabeling. Furthermore the half life must be a good match to the time between administration and imaging that is required to reach an adequate target/nontarget ratio that can vary over a wide range depending upon the application. For radiotherapy, radionuclides with longer half lives are often preferred if that results in delivering a higher radiation dose to the target. Nevertheless, an overriding prerequisite is always availability. Thus, in addition to its superior decay property, ^99m^Tc is often a favorite choice for planar and SPECT imaging since it may be made available on demand from a ^99^Mo/^99m^Tc radionuclide generator.

**Table 1:** Properties of selected metallic radionuclides useful in nuclear medicine.

Radionuclide	half-life	energy (KeV)	emitter	source
^64^Cu	12.7 h	653	β^+^	cyclotron
^67^Ga	78.3 h	93,185	γ	cyclotron
^89^Sr	50.6 d	1460	β^-^	reactor
^90^Y	64.1 h	2270	β^-^	reactor
^99m^Tc	6.02 h	141	γ	generator
^111^In	67.9 h	171,247	γ	cyclotron
^153^Sm	46.3 h	702,810;103	β^-^,γ	reactor
^177^Lu	6.7 d	176,497;113,208	β^-^,γ	reactor
^186^Re	90.6 h	936,1070;137	β^-^,γ	reactor
^188^Re	16.9 h	1500;155	β^-^,γ	generator
^201^Tl	73.1 h	135,167	γ	cyclotron

## 4. Chelators and/or Linkers

The labeling strategy of oligomers is very similar to that of other biologicals such as peptides and antibodies. If the radionuclide is an isotope of a metal, a chelator, possibly attached via a linker to avoid steric hindrances, is required. We describe below the use of MAG_3_, DTPA, and DOTA as bifunctional chelators (*i.e.,* with two functionalities, one permitting covalent attachment and another permitting chelation) because of their common use for radiolabeling oligomers with some metallic radionuclides.

### 4.1. MAG_3_ derivatives

When labeled with ^99m^Tc, mercaptoacetyltriglycine (MAG_3_) is a clinical radiopharmaceutical for imaging kidney function [14]. The sulfhydryl group in the clinical MAG_3_ radiopharmaceutical is protected by a benzoyl group that requires high temperatures and basic pH conditions for deprotection.

**Figure 2:**
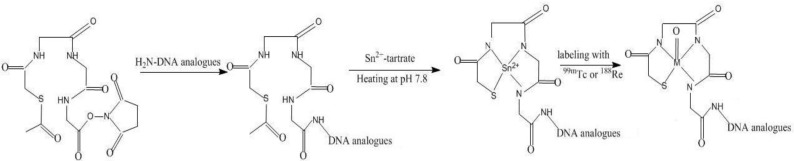
Conjugation of the NHS-MAG_3_ to amine-derivatized oligomers, and radiolabeling.

Because of its ability to stabilize chelate ^99m^Tc, MAG_3_ has also been modified into a bifunctional chelator for the labeling of biologicals. A NHS (N-Hydroxyl succinimide) activated MAG_3_ bifunctional chelator (S-acetyl NHS-MAG_3_) has been used for the labeling of amine derivatized oligomers with ^99m^Tc (Figure 2). To avoid the harsh conditions of boiled water temperature and alkaline pH of benzoyl deprotection, S-acetyl NHS-MAG_3_ was synthesized in which an acetyl replaces the benzoyl group. As a better leaving group, acetyl can be more easily removed at neutral pH and room temperature [15]. However, while the labeling of DNAs in this manner with ^99m^Tc at room temperature and neutral pH became routine [16,17,18,19], post-labeling purification was required to raise the radiochemical purity to 90% or higher [20]. Fortunately, oligomers tend to be insensitive to heat. In an investigation to examine the reasons for the lower labeling efficiency, the conjugation and labeling chemistry was found to be more complicated than expected. When the free S-acetyl NHS-MAG_3_ was labeled at room temperature, the product was an unidentified labeled product and not the expected ^99m^TcO-MAG_3_ [21] although the unidentified labeled products was converted into ^99m^TcO-MAG_3_ after heating. When NHS-MAG3 was first used to radiolabel an anime-derivatized MORF, low labeling efficiency was found despite boiling water temperatures [22,23]. Subsequent investigations found that the low labeling efficiency was not due to incomplete purification of the MAG_3_-MORF after conjugation but due to the labeling of impurities [20]. Liu et al found that these impurities could be removed by introducing a preliminary purification procedure before labeling and a labeling efficiency of over 95% is now obtained routinely [24,25].

To our knowledge, activated groups other than NHS active esters have not been used for the conjugation of MAG_3_ to oligomers. However, as earlier mentioned, all other conjugation approaches employed in the labeling of peptides and antibodies can be potentially translatable to the labeling of oligomers, for example MAG_3_ activated by an isothiocyanate group [26]. Like most chelators useful with ^99m^Tc, MAG_3_ can also form stable complex with ^186^Re and ^188^Re, because of similarities in the chemistry of technetium and rhenium.

### 4.2. DTPA

**Figure 3:**
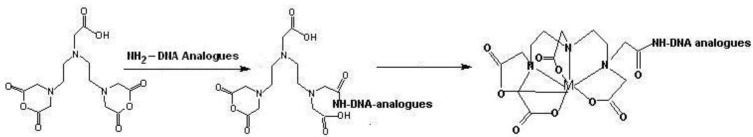
Conjugation of the cyclic DTPA anhydride to amine-derivatized oligomers, and radiolabeling.

Although the active DTPA ester formed *in situ* by reacting with a carbodiimide such as EDC [1-Ethyl-3(3-dimethylaminopropyl)carbodiimide Hydrochloride)] has been used for the conjugation of DTPA to biologicals [27], DTPA in the form of a bifunctional chelator has proved to be more popular. As the simplest bifunctional form of DTPA, the cyclic DTPA anhydride has been widely used [28], for example to radiolabel amine derivatized DNAs and MORFs with ^99m^Tc [19,27]. However unlike ^99m^Tc-MAG_3_, the ^99m^Tc within the DTPA chelator is susceptible to oxidation once the excess Sn(II) used in the labeling is removed [29]. Furthermore, the labeling efficiency is usually low because DTPA is a poor chelator for ^99m^Tc [30]. As such, DTPA has seen more use for attaching trivalent metals such as ^111^In as shown in Figure 3 [31] and radioactive lanthanides such as ^90^Y [32]. The labeling can be achieved by simply mixing the DTPA-conjugated biologicals with the acetate, the kinetics is rapid and an almost quantitative labeling efficiency is usually achieved even at neutral pH and room temperature. This method of labeling is therefore suitable for biologicals sensitive to excessive heat and acidity.

Earlier there was a concern that the use of the cyclic DTPA anhydride could cross link two biologicals because of the two anhydride groups [33]. However, to our knowledge, no direct evidence of cross linking has been reported. A plausible explanation is that the cyclic DTPA anhydride is normally added at a five-fold molar excess or higher. The excess anhydride is also often necessary to compensate for the hydrolysis of the anhydride that will occur in aqueous solution in competition with conjugation.

Another concern to the use of the cyclic anhydride is the possible compromised chelation stability because of the loss of one carboxylate to the conjugation [34,35]. Therefore other activated conjugation groups have been introduced in which the linker is attached to one of the ethylene carbons thus leaving all five carboxylates intact. For example, an isothiocyanate group has been added to the DTPA structure and used to conjugate biologicals [34] including RNA analogs [36]. The isothiocyanate benzyl DTPA is commercially available, the conjugation condition is mild, and the labeling efficiency is high for some isotopes, such as ^111^In, ^90^Y and ^177^Lu [37,38,39].

### 4.3. DOTA

**Figure 4:**
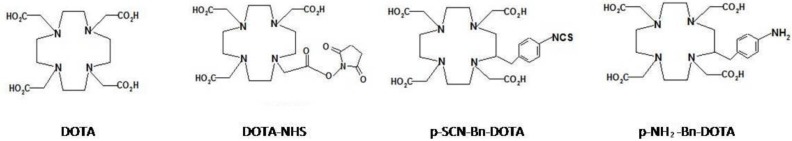
DOTA and three bifunctional DOTA chelators.

Similar to DTPA, 1,4,7,10-Tetraazacyclododecane-N,N’,N’’,N’’’-tetraacetic acid (DOTA) is also a good chelator for trivalent metals, such as In^3+^, Y^3+^ and other metals of the lanthanide series [40] and can be readily modified into a bifunctional chelator, although by first activating a carboxylate with a carbodiimide as with DTPA, DOTA itself may be conjugated. Alternatively, DOTA bifunctional chelators may be prepared by derivatization of one of the carboxylates, and their NHS or SCN activated derivatives as shown in Figure 4.

**Figure 5:**
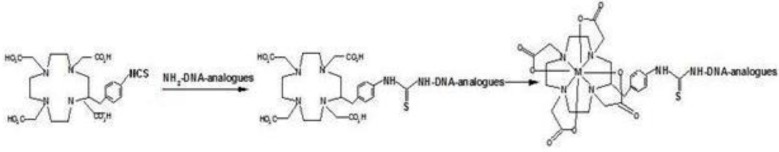
Conjugation of the SCN-benzyl-DOTA to amine-derivatized oligomers, and radiolabeling.

Unlike DTPA, DOTA is a macrocyle with a 12-membered tetraaza macrocycle ring and due to the rigid ring structure, its radiometal complexes tend to be kinetically inert to dissociation and its complexes are also characterized by high thermodynamic stability [41]. However, because the kinetics of radiolabeling of all macrocycles tends to be slow at room temperature, heating is often applied [42]. In addition, the ring size is a sensitive parameter of radiolabeling efficiency and radiolabel stability. For example, DOTA prefers smaller ions including Y^3+^ and other lanthanides to In^3+^ and Ga^3+^ [43]. A MORF has been labeled with ^90^Y and ^111^In using SCN-benzyl-DOTA [44]. Figure 5 illustrates conjugation of SCN-benzyl-DOTA to an amine-derivatized oligomer and the subsequent radiolabeling.

### 4.4. HYNIC

The bifunctional chelator 6-Hydrazinopridine-3-carboxylic acid (Hynic) is used mainly for radiolabeling of biologicals with ^99m^Tc [31,45,46]. Strictly speaking, Hynic is not a chelator because the strong association between Hynic and ^99m^Tc is not due to chelation but due to the formation of a single strong diazenido double bond. The oxidation state of technetium in this structure is 5 [^99m^Tc(V)] [47,48,49,50,51,52,53,54]. Although the ^99m^Tc bond to Hynic is very stable towards dissociation, it is susceptible to air oxidation. While chelators for ^99m^Tc are usually translatable to radiorhennium, Hynic may be an exception [55]. It has been reported that the labeling efficiency of a ^188^Re labeled Hynic conjugate dropped from 97% to 80% in 1 h upon storage [56], probably because rhenium is more easily air oxidized especially in the absence of excess tin (II).

**Figure 6:**
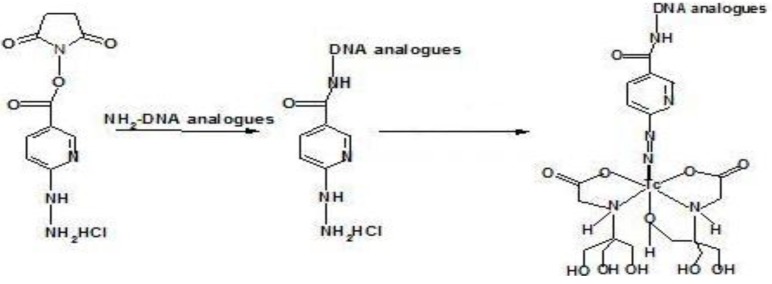
Conjugation of the NHS-Hynic to amine-derivatized oligomers, and radiolabeling with ^99m^Tc.

Because Hynic occupies only one or two coordination positions (see below) of the octahedral coordination sphere of technetium, a coligand is required. The wrong choice of coligands can seriously decrease the labeling efficiency and stability of the radiolabel. If a weak coligand such as tricine [57,58,59,60,61,62,63] (Figure 6) or glucoheptonate [64,65,66,67,68,69,70] is selected, dissociation of the coligand can occur *in vivo* and replaced with endogenous proteins leading to high normal tissue backgrounds [71]. Stronger coligands such as EDDA and tricine/phosphine can be introduced to replace tricine after labeling to avoid this ligands exchange [72,73].

The bifunctional chelator NHS-Hynic is commercially available and has been used to label oligomers but postlabeling purification was required [19,31]. In addition to labeling with ^99m^Tc(V), Hynic has also been used as a true bidentate chelator when used with ^99m^Tc(I) as ^99m^Tc(CO)_3_^+^, however the labeling chemistry remains poorly understood. One concern over this approach is high kidney accumulation that may be explained by the one remaining labile position [74].

## 5. Biological Properties and Labeling

Radiolabeled biologicals used in nuclear medicine are usually required to faithfully trace the behavior of the unlabeled biological *in vivo*. Since determining the biodistributions accurately of unlabeled compounds is extremely difficult, the reasonable assumption is usually made that the biodistribution of a biological will be unchanged if labeled by isotope substitution in which a stable atom in the structure is replaced with its radioactive isotope as mentioned above. However, as already mentioned, there are no convenient long lived, imaginable radioisotopes of naturally occurring elements such as carbon, oxygen and nitrogen. Biologicals are therefore usually radiolabeled with elements not found in biologicals such as technetium, indium and yttrium. Because the radioisotopes of these elements are metals, the modification of the biological usually requires attaching a relatively bulky chelator. For large molecules such as IgG antibodies, the effect on the biodistribution due to the radiolabeling is normally assumed to be minimal, however for molecules of small size such as peptides, biodistribution change after radiolabeling has been observed [75,76].

A search of the literature failed to find any comparison of biodistributions of oligomers before and after radiolabeling by chelation. It has been shown that biodistribution of an oligomer can be influenced by different conjugation groups and labeling methods [19]. However, the biodistributions of a 25 mer MORF oligomer labeled with ^90^Y and ^111^In using SCN-Benzyl-DTPA and with ^99m^Tc and ^188^Re using NHS-MAG_3_ were shown to be essentially identical [44]. This observation was partially supported by measuring the biodistributions of an 18 mer MORF oligomer labeled both with ^99m^Tc via MAG_3_ and ^111^In via DTPA. The biodistributions were again essentially identical in normal organs except for the intestinal tract [27] where the excretion was essentially negligible in the case of ^111^In and was about 2% in the case of ^99m^Tc.

## 6. Conclusions

Reports describing the radiolabeling of DNA and RNA and their analogs with radionuclides of diagnostic and therapeutic importance are continually appearing. However, we have focused herein only on the chelation labeling of oligomers with metallic radionuclides using common chelators popular in this and other laboratories. We hope that we have shown that a range of labeling methods are available for these biologicals to attach the more common metallic radionuclides useful in nuclear medicine but that each has its peculiar advantages and disadvantages. Compared to only a few years ago, these labeling methods may now be described as mature such that the emphasis need not be placed to the same degree on the labeling but on the use of these radiolabeled oligomers in various attractive *in vivo* applications.
